# Resistant and Refractory Obesity: The Complexity of Anti-Obesity Therapy Failure

**DOI:** 10.3390/ijms27062539

**Published:** 2026-03-10

**Authors:** Michał Nicze, Maciej Borówka, Adrianna Dec, Łukasz Bułdak, Aleksandra Bołdys, Bogusław Okopień

**Affiliations:** Department of Internal Medicine and Clinical Pharmacology, School of Medicine in Katowice, Medical University of Silesia, Medyków 18, 40-752 Katowice, Poland; michal.nicze@sum.edu.pl (M.N.); aboldys@sum.edu.pl (A.B.);

**Keywords:** anti-obesity therapy, incretins, liraglutide, microbiome, obesity phenotypes, pharmacogenomics, pharmacokinetics, semaglutide, tirzepatide, treatment resistance

## Abstract

Pharmacotherapy is a key component of obesity management, yet treatment failure remains a prevalent challenge in clinical practice. Such failure may present as insufficient pharmacological response, early discontinuation, or post-treatment weight regain, underscoring the discrepancy between clinical trial efficacy and real-world outcomes. The effectiveness of anti-obesity medications (AOMs) is influenced by psychiatric comorbidities, including depression, anxiety, and disordered eating patterns, as well as environmental and socioeconomic factors such as limited healthcare access, weight-related stigma, and high medication costs. Individual characteristics, including physical activity, body composition, visceral adiposity, and microbiome profile, further modulate treatment outcomes. Pharmacokinetic and pharmacotherapeutic limitations such as drug-phenotype mismatch, route of administration, suboptimal formulations, and exposure to counterfeit products also compromise efficacy. No less important are genetic and immunological factors, comprising pharmacogenomic variants of both incretin and melanocortin receptors along with antidrug antibodies (ADAs), which may constitute therapy resistance. Concomitant medications and comorbid endocrine disorders can additionally attenuate weight-loss effects. The objective of this review is to characterize the multifactorial nature of resistance and refractoriness to anti-obesity therapy, and the importance of identifying pretreatment predictive factors for recognizing individuals at risk of inadequate or lack of response, thereby enabling personalized management strategies and improving long-term clinical outcomes, particularly in “difficult-to-treat” patients.

## 1. Introduction

Since obesity and its life-threatening complications, such as type 2 diabetes mellitus (T2DM), hypertension, hyperlipidemia, metabolic dysfunction-associated steatotic liver disease (MASLD), and chronic kidney disease, have become a major burden for healthcare systems worldwide—with incidence rising rapidly particularly in developed countries [1]—appropriate management strategies must be implemented.

Among the available approaches, modern anti-obesity pharmacotherapy—especially the latest incretin-based agents acting on glucagon-like peptide-1 (GLP-1) receptors [2], as well as the single currently approved dual agonist that also targets glucose-dependent insulinotropic peptide (GIP) receptors [3]—has demonstrated a favorable safety profile and represents the most effective long-term method of reducing excess body weight, provided it is used in accordance with medical recommendations and combined with physical activity and a low-calorie diet [4]. Other therapeutic modalities, such as naltrexone/bupropion, orlistat or phentermine/topiramate, while less potent, seem to be an important approach in certain groups of patients. These therapeutic options are indicated for patients with a Body Mass Index (BMI) of at least 30 kg/m^2^, or for those with a BMI of 27 kg/m^2^ or higher who also present with comorbidities linked to obesity such as T2DM, hypertension, or sleep apnea [5].

Several factors may contribute to reduced treatment efficacy, including comorbidities such as mental disorders, socioeconomic determinants, environmental influences, pharmacokinetic variability, iatrogenic issues, and genetic or immunological predispositions. Equally significant can be measurable individual characteristics, for example pretreatment body composition or the intestinal microbiome.

According to the literature, baseline response rates, typically expressed as percentage or absolute reductions in body weight, differ significantly among AOMs. To date, tirzepatide, the only approved dual GIP/GLP-1 receptor agonist (RA), has demonstrated the highest efficacy in the SURMOUNT-5 trial, with weight reductions reaching up to 20.2% (95% confidence interval [CI]: 19.1–21.4) [6], similar to outcomes observed after bariatric surgery [7], compared with 13.7% (95% CI: 12.6–14.9) for GLP-1RA semaglutide (*p* < 0.001) after 18 months of once-weekly injections at maximal tolerated doses (10 mg or 15 mg vs. 1.7 mg or 2.4 mg, respectively) [6]. Another GLP-1RA, liraglutide, remains an effective alternative, with once-daily injections of 3.0 mg over one year resulting in a mean (±standard deviation [SD]) weight loss of 8% ± 6.7%, which was significantly greater than that observed with placebo (*p* < 0.001) [8]. The combination of phentermine and topiramate, administered daily at the highest approved dose (15/92 mg), consistently produced 9.8% (95% CI: 9.3–10.4; *p* < 0.0001) placebo-adjusted mean weight loss over 56 weeks [9], whereas naltrexone/bupropion (32 mg/360 mg daily) resulted in a significantly greater average weight loss of approximately 6.5% when compared to placebo (*p* < 0.001) over the same period [10]. Moreover, orlistat (120 mg three times daily) was associated with a mean weight reduction of approximately 10% at one year, with a sustained ~5% weight loss at four years, demonstrating significantly greater long-term weight reduction compared with placebo (*p* < 0.001) [11].

These data underscore that, while tirzepatide remains the most potent among currently approved AOMs, GLP-1RAs and the other aforementioned agents also provide clinically relevant, albeit smaller, reductions in body weight [12]. They may be considered viable options, particularly when patient-specific factors preclude the use of dual GIP/GLP-1RA. It is worth noting that in real-world settings, where strict trial inclusion and exclusion criteria are not applied, these weight reductions might be lower than those observed in randomized controlled trials (RCTs) [13].

As far as anti-obesity treatment failure is concerned, it is essential to distinguish between premature discontinuation of therapy by the patient and a true lack of pharmacological response to anti-obesity agents, as both may ultimately lead to weight regain. Non-adherence or early discontinuation reflects behavioral or practical barriers, including drug-related adverse effects (AEs), rather than intrinsic drug inefficacy, whereas the general definition of treatment non-response (resistance) typically refers to insufficient weight loss despite appropriate adherence and adequate dosing—usually defined as less than 5% weight loss (or less than 3% in diabetic patients) within the first three to six months of therapy [5,14].

In this context, it is essential to distinguish between resistant and refractory obesity, terms often used interchangeably despite representing distinct clinical entities. Resistant obesity is defined as the inability to achieve and maintain ≥5% weight loss over three months despite the use of ≥3 treatment modalities at maximal or maximally tolerated intensity. These modalities should include intensive behavioral therapy (IBT), medical nutrition therapy (MNT), and the most effective available GLP-1RA. In settings where GLP-1RAs are unavailable, resistant obesity may be diagnosed if IBT, MNT, and at least one approved AOM (e.g., orlistat, phentermine/topiramate, or naltrexone/bupropion) fail to achieve ≥5% weight loss over six months [15].

Refractory obesity, in contrast, represents a more severe phenotype, defined as failure to achieve and maintain ≥5% weight loss over six months despite ≥5 therapeutic modalities applied at maximal or maximally tolerated intensity. Required interventions include IBT, MNT, a GLP-1RA, a trial of a very low-calorie or low-calorie diet (VLCD/LCD), and at least one additional pharmacological agent (orlistat, phentermine/topiramate, or naltrexone/bupropion); in selected cases, bariatric procedures may also have been attempted [15].

Before establishing a diagnosis of resistant or refractory obesity, pseudo-resistance/pseudo-refractoriness must be excluded. This includes inadequate adherence to dietary, physical activity, or pharmacological recommendations; improper drug administration (e.g., incorrect injection technique for GLP-1RAs or inappropriate meal timing with orlistat); untreated comorbidities associated with weight gain or fluid retention (e.g., heart failure, renal or hepatic dysfunction, hypothyroidism); and concomitant use of weight-promoting medications such as glucocorticoids or high-dose insulin [15]. Distinguishing between resistant and refractory obesity provides a practical framework for assessing treatment adequacy, identifying non-adherence and iatrogenic contributors, and guiding therapeutic intensification. It also facilitates interpretation of pharmacogenetic variability and supports individualized management strategies in patients with complex, biologically driven obesity phenotypes.

The proportion of patients who fail to achieve the ≥5% weight-loss threshold varies by medication [5]. Tirzepatide, among drugs approved for clinical use, is associated with the lowest proportion of non-responders (approx. 9%) [3], followed by semaglutide (13.6%) [16], phentermine/topiramate (30%) [17], liraglutide (36.8%) [8], orlistat (approx. 40%) [18] and naltrexone/bupropion, which has the highest rate of non-response (52%) [19]. In contrast, the novel investigational molecule LY3437943 (retatrutide), a triple hormone receptor agonist targeting GLP-1, GIP, and glucagon receptors, administered once weekly at doses of 8 or 12 mg over 48 weeks, was reported to achieve a 100% response rate in preliminary results from a phase II trial [20], offering hope for obese patients who did not respond to currently available therapies. Importantly, the same trial demonstrated a mean body-weight reduction of 24.2% in the 12 mg dose group following dose escalation from an initial 2 mg dose (compared with −2.1% in the placebo group), exceeding the approximately 20% weight loss reported with tirzepatide. Table 1 summarizes the mechanisms of action, common AEs, and key clinical trial data—including overall efficacy, treatment failure, and discontinuation rates—of currently approved obesity therapies.

In light of these observations, the purpose of this paper is to highlight the multifactorial nature of resistance and refractoriness to anti-obesity therapy in the context of potential predictive factors that should be considered prior to treatment initiation in order to identify subjects at risk of a suboptimal therapeutic response or true non-response.

While many previous studies have examined mostly individual determinants of treatment failure, to the authors’ knowledge there is a lack of comprehensive and reliable frameworks integrating biological, genetic, immunological, pharmacological and psychosocial factors to guide practical clinical decision-making. This work aims to fill that gap by synthesizing current evidence and proposing an approach to identify “difficult-to-treat” patients, thereby providing a practical contribution to personalized obesity management. Recognizing these patients is critical for optimizing management by adjusting therapeutic strategies, providing additional support, and effectively improving long-term outcomes.

## 2. Methods

To explore resistance and refractoriness to anti-obesity therapy, a comprehensive literature search was conducted using the PubMed/MEDLINE, Scopus, and Google Scholar databases. The search covered publications from January 1990 to January 2026 and was restricted to articles published in English. The search strategy employed Medical Subject Headings (MeSH) and free-text terms, including combinations of “obesity”, “anti-obesity medications”, “treatment resistance”, “weight loss failure”, “refractory obesity”, “non-response”, “response predictors”, “drug immunogenicity”, “receptor polymorphisms”, “receptor mutations”, “gut microbiota”, “environmental factors”, and “psychiatric comorbidities”.

The initial search yielded approximately 14,500 records. After removal of duplicates and screening of titles and abstracts, almost 1200 articles were selected for full-text review based on relevance to pharmacological treatment failure, resistance, or heterogeneity of response in obesity management. Moreover, particular study records on ClinicalTrials.gov. were searched and selected. Ultimately, 234 publications—including RCTs, observational studies, post hoc analyses, and relevant review articles—were included in the final analysis.

Articles were selected based on clinical relevance, methodological quality, and contribution to understanding biological, genetic, immunological, clinical, and psychosocial factors associated with inadequate or absent response to anti-obesity therapy. No formal meta-analysis was performed due to heterogeneity in study designs, outcome definitions, and therapeutic agents.

## 3. Determinants of Anti-Obesity Pharmacotherapy Effectiveness

A. Primary resistance to therapy

Pharmacotherapy may fail because of impaired signaling resulting from receptor damage or genetic variants, inadequate drug absorption, or mechanisms that neutralize therapeutic activity, such as the development of ADAs. Individual genetic predisposition is among the most difficult factors to predict with regard to AOM efficacy, and this relationship constitutes the central focus of pharmacogenetics.

### 3.1. The GLP-1 Receptor (GLP1R) Gene Polymorphism

*GLP1R* gene polymorphism influences glycemic control, the glucose-dependent insulin response, and overall insulin sensitivity. Furthermore, it plays a role in determining anthropometric parameters, such as BMI, waist circumference and obesity predisposition [29].

GLP-1R currently serves as a primary drug target for obesity and T2DM treatment and its genetic diversity has become the subject of many studies as a potential cause of differential response to therapy. These variants encompass several functional alterations, including impaired surface expression, loss-of-function (LoF), and gain-of-function (GoF) mutations [30].

A genetic association study of 200,000 UK Biobank participants identified reduced *GLP1R* expression on the cell surface due to loss-of-function mutation as a distinct risk factor for diminished glucose metabolism and higher adiposity, characterized by elevated glycated hemoglobin (HbA1c) and BMI [30]. In the study by Michałowska et al., the impact of the *GLP1R* single-nucleotide polymorphisms rs2268641 and rs6923761 was examined in a Polish cohort. The researchers found that AA carriers of rs6923761 had a higher risk of excessive body weight, whereas TT carriers of rs2268641 exhibited a lower risk [31]. Furthermore, the EPOCH study indicated that individuals with the homozygous GG variant exhibit a faster increase in BMI during childhood and adolescence, as well as higher insulin resistance [29]. A study published in October 2025 observed that the *GLP1R* rs6923761 variant promotes weight loss after liraglutide treatment while worsening its other metabolic effects [32].

The negative influence of genetic polymorphism on the effectiveness of GLP-1RAs in reducing body weight was proven, among others, in a study including 57 women with obesity and polycystic ovary syndrome (PCOS). They were genotyped for common *GLP1R* single-nucleotide polymorphisms rs6923761 and rs10305420. Each patient received 1.2 mg of liraglutide for 12 weeks (as the only weight-loss medication), supplemented by a calorie deficit of 500–800 kcal per day and at least 30 min of daily moderate-intensity physical activity. The *GLP1R* rs10305420 polymorphism was linked to a poor anti-obesity response to liraglutide (*p* = 0.025). On the other hand, carriers of at least one rs6923761 minor allele showed a tendency toward greater weight loss (*p* = 0.058). Additionally, reductions in glucose levels—both at fasting and during the oral glucose tolerance test—were comparable between the two groups [33]. Moreover, in the population of obese Chinese individuals, the presence of the T allele was associated with less weight loss (about 1.27 kg less) and a smaller reduction in HbA1c after 6 months of exenatide treatment [34].

Since genetic variations in the *GLP1R* gene affect how patients respond to GLP-1RAs, pharmacogenetic screening can facilitate personalized treatment strategies in the future. In Table 2, we summarized selected studies on *GLP1R* polymorphisms and their impact on body weight as well as the effects of GLP-1RAs. Currently, there are no recommendations on phenotyping of GLP-1R prior to the initiation of therapy, but potential assessment might be considered in future in patients with primary resistance.

### 3.2. The Melanocortin 4 Receptor (MC4R) Gene Mutations

Certain types of severe early-onset obesity result from genetic mutations that impair the melanocortin pathway, a critical regulator of body metabolism. MC4R mediates feeding behavior within the hypothalamus, acting as a critical component in appetite control. After food intake, leptin receptors (LEPR) are stimulated and secrete proopiomelanocortin (POMC), which is converted by proprotein convertase subtilisin-kexin type 1 (PCSK1) into melanocortin peptides, direct activators of MC4R, leading to a decrease in appetite. Genetically determined disruption of the mentioned pathway at any stage—LEPR/POMC/PCSK1 deficiency—causes significant clinical implications, including the occurrence of hyperphagia [35]. More than 100 distinct mutations have been identified within the human *MC4R* gene sequence—it is the most common genetic obesity cause and heterozygous LoF mutations are the most frequently identified, reported in 2–5% of individuals with early-onset severe obesity [36]. Two studies indicated that children carrying the *MC4R* mutation face significant challenges in weight management. In these individuals, conventional interventions-such as caloric restriction and increased physical activity-yield diminished results. Furthermore, achieving sustainable weight loss is notably more difficult, as these patients exhibit a higher predisposition to weight regain compared to those without [37,38]. GLP-1RAs, including the dual GIP/GLP-1RA tirzepatide, may seem to be a therapeutic alternative for this group of patients. In the SURMOUNT-1 clinical trial, 32 out of 2291 participants were identified as carriers of the *MC4R* mutation. After 72 weeks of treatment with tirzepatide, comparable weight loss was observed in both cohorts: carriers of the *MC4R* mutation achieved an 18.3% weight reduction, compared to 19.9% in non-carriers [36]. Additionally, the first MC4R agonist approved for the treatment of monogenic obesity has entered the market. Setmelanotide promotes satiety and weight loss in patients with obesity caused by rare POMC/MC4R pathway mutations. From February 2017 to September 2018, 10 patients joined the POMC trial and 11 joined the LEPR trial. After approximately one year of treatment, 80% of POMC participants and 45% of LEPR lost at least 10% of their initial body weight [35]. Despite promising results, further research is needed to determine if setmelanotide is effective for weight reduction in patients with more prevalent, polygenic types of obesity.

### 3.3. Drug Immunogenicity

The ability of drugs, particularly peptides, to induce an immune response and stimulate ADA formation is a key factor when considering potential AEs on pharmacokinetics, as well as drug efficacy and safety. This possibility has also been explored regarding currently available AOMs.

GLP-1RAs, which are analogs of endogenous human GLP-1, exhibit lower immunogenicity than alternatives like exendin-4 (derived from the saliva of the *Gila monster*). Generally, a higher degree of homology with native GLP-1 correlates with a reduced risk of developing ADAs. Liraglutide shares 97% sequence homology with the human peptide, while semaglutide shows 94% and exenatide 53% homology [39]. This was evaluated in the LEAD study, which assessed the immunogenicity of liraglutide and exenatide. After 26 weeks, ADAs were detected in 8.7% (32/369) and 8.3% (49/587) of patients receiving liraglutide at doses of 1.2 mg and 1.8 mg, respectively; these antibodies did not diminish the HbA1c-lowering effect. Conversely, in the LEAD-6 trial, 61% of patients (113/185) developed anti-exenatide antibodies and high ADAs levels, and this was accompanied by reduced HbA1c-lowering potential (*p* = 0.0022) [40]. Across the SUSTAIN studies ADAs occurred in only 1.4% of semaglutide-treated patients and were not associated with any changes in clinical efficacy or safety [39].

In the case of tirzepatide, the presence and effect of ADAs were assessed in the SURPASS studies at baseline, throughout the study period, and at the endpoint defined as 40 (SURPASS-1,-2,-5) or 52 week (SURPASS-3, -4, Japan-Mono, and Japan-Combo). During treatment, antibody development was observed in 51.5% of patients receiving the drug, and their maximum titers ranged from 1:20 to 1:81,920. Neutralizing antibodies (NAbs) that target the activity of tirzepatide on GIP and GLP-1 receptors were detected in 1.9% and 2.1% of patients, respectively. Cross-reactive NAbs against native GIP or native GLP-1 were detected in less than 1.0% of patients. Ultimately, it was shown that their presence affected neither the pharmacokinetics nor the efficacy of the drug. However, ADAs were associated with a higher incidence of hypersensitivity and injection site reactions compared to the group of patients who did not develop them [41].

While the induction of NAbs in modern peptide-based drugs are low, a rapid loss of therapeutic efficacy after initial good impact on body weight may be a resultant of ADAs. Two options seem reasonable at this point, (1) to assess the titer of ADAs and in case of high level change the therapeutic method—availability of this route is limited, the other (2) is to empirically switch one GLP-1RA to another. Both of them rely on the fact that cross-action of the ADAs will not affect drug efficacy.

### 3.4. Pharmacological Issues

Pharmacological failure in obesity management does not invariably indicate insufficient efficacy observed in clinical trials but often can result from challenges encountered in real-world pharmacokinetic conditions and therapeutic implementation. Such challenges may arise at several levels, including suboptimal drug selection, limitations related to the route of administration, and issues concerning pharmaceutical formulation quality, ultimately resulting in inadequate therapeutic exposure.

#### 3.4.1. Route of Administration and Formulation

The route of administration and formulation of peptide-based incretin therapies pose additional challenges that may affect both treatment efficacy and patient adherence. Therapeutic peptides such as GLP-1RAs are inherently susceptible to enzymatic degradation and exhibit poor absorption in the gastrointestinal tract. Consequently, the oral bioavailability of most peptide drugs remains extremely low without specialized delivery systems, necessitating parenteral administration to achieve therapeutic plasma concentrations [42]. This reliance on subcutaneous injections may reduce patient adherence due to perceived inconvenience or injection-related anxiety, potentially resulting in inconsistent drug exposure and treatment resistance, similar to the fear of insulin injections observed in some patients with T2DM [43]. Moreover, injectable administration may limit the practicality of GLP-1RAs in certain populations, particularly elderly individuals with visual, motor, or cognitive impairments [44].

Even for the only currently marketed oral peptide formulation (semaglutide co-formulated with the absorption enhancer salcaprozate sodium [SNAC]) systemic bioavailability remains low (approx. 1–2%) [42]. Effective drug absorption therefore requires strict adherence to specific dosing instructions, including once-daily administration in the fasting state, ingestion with a small volume of water, and delaying food or other medications afterward. These requirements may further reduce acceptability and adherence in some patients. In addition, tablet splitting of higher-dose formulations to prolong treatment duration might possibly compromise dosing accuracy and limit therapeutic efficacy as well.

#### 3.4.2. Quality Issues Related to Substandard and Counterfeit Medicines

The presence of substandard and counterfeit AOMs in global markets represents another significant pharmacokinetic and safety concern. Falsified weight-loss products frequently contain incorrect, absent, or inconsistent amounts of active pharmaceutical ingredients and may be manufactured without adherence to regulatory quality standards, resulting in inaccurate dosing, unpredictable pharmacokinetics, and potential public health hazard [45]. AOMs are particularly prone to falsification in unregulated channels, such as online marketplaces, and these “cheaper” counterfeit formulations purchased without a prescription are not only ineffective but can also cause severe AEs, including hypoglycemic shock or coma, suggesting that some may even contain insulin instead of GLP-1RAs [46]. This phenomenon may further erode patient confidence in pharmacotherapy, contribute to treatment discontinuation, and ultimately compromise long-term obesity management outcomes.

#### 3.4.3. Mechanism-Drug Mismatch

Pharmacokinetic limitations associated with mechanism-drug mismatch seem to be an underappreciated contributor to anti-obesity treatment failure. Variability in treatment response can occur when the pharmacological action of a chosen agent does not align with the predominant pathophysiological background of a particular patient’s obesity phenotype. Recognition of the pathophysiological heterogeneity of obesity has led to the description of distinct phenotypes, commonly categorized as “hungry brain” (impaired satiation), “hungry gut” (impaired satiety), “emotional hunger” (reward-driven eating), and “slow burn” (reduced metabolic rate). This conceptual framework provides a basis for more individualized treatment selection, with GLP-1RAs preferentially targeting satiety-related phenotypes, naltrexone–bupropion addressing reward-based eating behaviors, and dual incretin therapy (tirzepatide) potentially benefiting patients with metabolic inefficiency. This phenotype-driven strategy demonstrated promising results. Patients who received treatment tailored to their phenotype achieved significantly greater weight loss after 12 months compared to those receiving standard care (15.9% vs. 9.0%, respectively). Importantly, current data suggest that outcomes of bariatric surgery may also vary across phenotypic subgroups, indicating that phenotype-guided approaches could optimize both pharmacological management and surgical decision-making [5,47,48].

Pharmacokinetic issues and counterfeit compounds require candid discussions with patients regarding the anxiety for a specific drug delivery method (i.e., injections—less convenient but reliable administration, oral—more convenient but may affect serum concentration) and hazards associated with drugs of unknown origin. The final paragraph of this section deals with an essential topic, which is patient-based selection of therapeutic modality, instead of drug-centered one. Background and recommendations in those cases will be provided in the following sections.

#### 3.4.4. The Influence of Microbiota on the Effectiveness of Pharmacotherapy

It is known that the composition of the intestinal microbiota may influence the effectiveness of specific interventions, including the effectiveness of pharmacotherapy. Regarding the impact of microbiota on the effectiveness of pharmacological weight-loss treatments, data are scarce. However, it is worth mentioning that the influence of gut microbiota composition on the effectiveness of GLP-1RAs in lowering blood glucose in patients with T2DM has been assessed [49].

B. Psychosocial, clinical, and patient-related determinants of treatment response

Lack of weight reduction during lifestyle interventions augmented by pharmacotherapy may result from a complex interplay of psychosocial, clinical, and patient-related factors that influence treatment response. These determinants are addressed in detail in the subsequent sections.

### 3.5. Coexisting Psychiatric Disorders

Psychiatric comorbidities—including depression, anxiety, and disordered eating patterns such as emotional eating (EE), binge eating disorder (BED), and night eating syndrome (NES)—substantially modulate the response to anti-obesity therapy. Their impact reflects not only behavioral factors affecting adherence, but also shared biological mechanisms involving inflammation, neuroendocrine dysregulation, and altered reward processing, which may be further compounded by the psychiatric effects of concomitant psychotropic or certain AOMs.

#### 3.5.1. Depression and Anxiety

Depression and obesity frequently co-occur through overlapping pathophysiological pathways, with each condition increasing vulnerability to the other [50]. Excess adipose tissue promotes chronic low-grade inflammation, leptin resistance, and cytokine-mediated disruption of monoaminergic neurotransmission, all of which are implicated in depressive symptomatology [50,51]. In parallel, stress-related hyperactivation of the hypothalamic–pituitary–adrenal (HPA) axis and hypercortisolemia favor visceral adiposity, while sleep disturbances with elevated ghrelin further exacerbate metabolic dysregulation [50]. These biological alterations interact with emotional dysregulation and maladaptive coping behaviors, reinforcing the bidirectional relationship between depression and obesity [52]. Atypical depression, characterized by hyperphagia and weight gain, confers particularly high obesity risk [53,54,55].

Anxiety disorders show a similar bidirectional association with obesity, driven by chronic stress exposure, neuroendocrine dysregulation, and weight-related stigma [53,56,57]. Social anxiety is especially linked to obesity, as fear of stigma and social withdrawal amplify emotional dysregulation and promote maladaptive eating behaviors [58]. From a mechanistic perspective, sustained anxiety-related activation of stress pathways may worsen insulin resistance and favor treatment resistance.

Integrated management combining behavioral weight control, cognitive–behavioral therapy (CBT), and lifestyle interventions is therefore recommended for both depression and anxiety [52,59,60,61,62]. Pharmacotherapy remains an important adjunct: selective serotonin reuptake inhibitors (SSRIs) are first-line agents [52], while BMI may inform antidepressant selection, as patients with severe obesity show greater symptom improvement with bupropion-based regimens [63]. Fluoxetine has additionally demonstrated modest weight reduction alongside antidepressant efficacy [64]. Among AOMs, naltrexone/bupropion and phentermine/topiramate are associated with increased anxiety-related AEs, whereas tirzepatide and GLP-1RAs show no consistent anxiety signal in RCTs [65], despite conflicting observational data for GLP-1RAs [66]. These findings highlight the need for individualized psychiatric assessment when selecting pharmacotherapy.

#### 3.5.2. Eating Disorders

Disordered eating in obesity reflects dysfunction of neural circuits governing stress responsiveness, reward valuation, and inhibitory control [52,67]. EE represents a behavioral interface between affective psychopathology and obesity, characterized by consumption of highly palatable foods in response to negative emotions [68,69,70,71,72]. At the molecular level, chronic stress may induce HPA-axis hypoactivity, shifting energy intake toward hedonic rather than homeostatic regulation [69,73].

BED, the most prevalent eating disorder in obesity, is defined by recurrent loss-of-control overeating without compensatory behaviors [53]. Its neurobiology implicates dopaminergic and opioid reward pathways, impaired inhibitory control, and genetic susceptibility [74], overlapping with genetic risk of attention-deficit/hyperactivity disorder (ADHD) [75] and impulse-control disturbances seen in borderline personality disorder [76]. NES represents a related phenotype marked by circadian misalignment of food intake and altered reward signaling, frequently accompanied by depressive symptoms [77,78,79].

Given these shared mechanisms, interventions targeting emotional regulation and reward processing are more effective than caloric restriction alone, which may exacerbate compensatory overeating [69]. CBT consistently shows the greatest efficacy among psychological interventions for EE and BED-related weight outcomes [80,81,82,83,84,85,86]. Pharmacotherapies acting on central neurotransmitter systems—including atomoxetine (a selective noradrenaline reuptake inhibitor), lisdexamfetamine (a prodrug of amphetamine and the only approved pharmacotherapy for BED), bupropion–naltrexone, and phentermine/topiramate—reduce binge frequency and craving by modulating catecholaminergic, opioid, and GABAergic–glutamatergic pathways [87,88,89,90,91,92]. GLP-1RAs additionally attenuate hedonic eating through hypothalamic and mesolimbic mechanisms, with semaglutide showing particular promise in BED [93,94,95,96]. In contrast, agents lacking central effects, such as orlistat, do not improve eating-disorder psychopathology and may be ineffective in this population [97].

#### 3.5.3. Clinical Implications

Across depressive, anxiety, and eating-disorder spectra, psychiatric comorbidities converge on shared molecular disturbances involving inflammation, stress-axis dysregulation, reward-system alterations, and impaired impulse control, all of which can undermine the response to obesity treatment. Effective obesity management may alleviate comorbid psychiatric symptoms, while adequate treatment of mood and anxiety disorders can, in turn, enhance weight-loss outcomes. Conversely, untreated or insufficiently managed psychopathology may reduce the efficacy of anti-obesity interventions, increase the risk of weight regain, and compromise adherence. These interdependencies underscore the need for early identification through systematic psychiatric screening in patients with obesity (e.g., using validated mood assessment tools such as the Beck Depression Inventory), as well as a multidisciplinary approach integrating psychotherapeutic interventions with individualized selection of AOMs—preferably those targeting appetite regulation, reward processing, and emotional control rather than inappropriate or ineffective therapies (e.g., orlistat). In selected patients, combination pharmacotherapy may further enhance efficacy by addressing multiple interacting pathophysiological pathways, thereby improving long-term outcomes.

### 3.6. Environmental and Socioeconomic Factors

Environmental and socioeconomic determinants substantially limit the effectiveness of anti-obesity treatment by interacting with biological, behavioral, and pharmacological mechanisms. Weight-related stigma, social isolation, healthcare system deficiencies, and economic barriers reduce access to care and adherence while activating stress-related pathways that promote metabolic dysregulation and weight regain. These factors frequently act synergistically, reinforcing obesogenic behaviors and limiting long-term treatment success.

#### 3.6.1. Weight-Related Stigma and Social Isolation

Weight-related stigma is a key contributor to obesity treatment failure. Stigmatization and discrimination, including in healthcare settings, are associated with psychological distress, healthcare avoidance, and poor adherence to lifestyle and pharmacological interventions [98]. Chronic exposure to stigma activates stress-responsive neuroendocrine pathways, which may exacerbate appetite dysregulation, weight gain and insulin resistance, reinforcing a vicious cycle of obesity [99]. Social isolation and loneliness are also more prevalent in individuals with obesity and are linked to poorer dietary behaviors and reduced treatment engagement [100,101]. Stigma-aware care and psychosocial support may therefore mitigate stress-related biological effects and improve adherence.

#### 3.6.2. Healthcare Systems Failures

Despite available guidelines, obesity remains underdiagnosed and undertreated due to limited clinician training, low confidence, and persistent weight bias among healthcare professionals [102,103,104]. These shortcomings impair continuity and quality of care and reduce long-term adherence [105,106]. Human coronavirus disease 2019 (COVID-19)–related healthcare disruptions further limited access to treatment and contributed to weight gain and poorer adherence, with effects persisting beyond the acute pandemic phase [107,108,109]. Improving obesity treatment outcomes requires systematic recognition, clinician education, bias reduction, and maintenance of care continuity, including appropriate use of telemedicine.

#### 3.6.3. Negative Social Support

Although social support generally improves weight-management outcomes [110,111], adverse interpersonal dynamics such as sabotage, feeding, and collusion can undermine obesity treatment [112]. Sabotage entails discouraging healthy behaviors, whereas feeding involves promoting food intake, particularly within close relationships [112,113]. Collusion, defined as avoidance of addressing maladaptive behaviors, can occur in both social and clinical contexts. Together, these patterns of negative support contribute to anti-obesity treatment failure [112,114,115]. Clinicians should therefore screen for sabotage, feeding, and collusion and, when appropriate, involve family members in education and counseling to improve adherence and long-term outcomes.

#### 3.6.4. Socioeconomic Barriers

High costs and inconsistent reimbursement limit access to effective anti-obesity pharmacotherapy. Modern incretin-based agents, such as semaglutide and tirzepatide, often cost hundreds to over a thousand dollars per month without insurance coverage, restricting use among socioeconomically disadvantaged patients despite strong efficacy [116]. In the United States, Medicare excludes AOMs, and many insurers impose restrictive eligibility criteria [117]. In contrast, NHS England’s phased tirzepatide rollout, which prioritizes patients based on comorbidity and integrates lifestyle support, offers a more equitable model [118]. Rising non-medical demand further strains supply, exacerbating disparities and increasing treatment discontinuation [44,119]. Expanding coverage, implementing income-based subsidies, and prioritizing medical indications may improve equitable access and treatment durability.

#### 3.6.5. Safety Concerns Related to Incretin-Based AOMs

Despite their proven efficacy, novel incretin-based AOMs can be limited by AEs that contribute to treatment discontinuation or reluctance to initiate therapy. RCTs of semaglutide and tirzepatide consistently demonstrate that the most common AEs are gastrointestinal—typically nausea, diarrhoea, vomiting, and constipation. These events are generally mild to moderate and transient but still lead to discontinuation in a subset of patients [16,120].

Beyond these recognized AEs, concerns about potential long-term oncological risks associated with GLP-1RAs, particularly medullary thyroid cancer and kidney cancer, have raised apprehension among patients and clinicians. Experimental and post-marketing data suggest a possible association, and although no causal link has been established [121,122], such findings may reinforce patient hesitancy toward GLP-1RA therapy.

Taken together, both actual AEs and perceived long-term safety concerns may therefore limit acceptance, adherence, and overall effectiveness of incretin-based therapies regardless of their high costs. However, thorough physician counselling may reduce patient anxiety for the AEs, while gradual dose escalation, close monitoring, and supportive follow-up may further enhance tolerability, ultimately improving treatment persistence

### 3.7. Individual Factors

#### 3.7.1. Age

According to statistical data from the European Union member states, the prevalence of obesity among adults increases with age, with the exception of the oldest age group [123]. However, the management of excess body weight in older patients is influenced by several additional factors, notably a higher incidence of comorbidities [124] and the consequent use of polypharmacy, which may attenuate the effectiveness of anti-obesity therapies. This is further complicated by the phenomenon of the obesity paradox, where older patients with overweight or mild obesity exhibit a lower all-cause mortality rate [125,126]. Therefore, to enhance the effectiveness of obesity management across specific age groups, it is essential to review studies that evaluate the influence of age on treatment outcomes.

Regarding behavioral interventions, the results from various studies are not entirely consistent. For instance, the study conducted by Leyden et al. found no significant effect of age on the percentage of excess body weight loss achieved [127]. Similar findings were reported in a study evaluating online behavioral interventions, where also no correlation was established between participants’ age and the degree of weight loss achieved [128]. Conversely, the study conducted by Svetkey et al. reported superior outcomes from behavioral methods of obesity treatment in the cohort of patients aged over 60 years [129]. Interestingly, in the pediatric population, superior treatment outcomes have been observed in the younger age group [130].

Furthermore, some studies exhibit variations regarding the outcomes of surgical treatment. For instance, Major et al. observed a greater percentage loss of excess body weight and excess BMI in patients younger than 50 years [131]. Similarly, Contreras et al. also observed a greater percentage reduction in excess BMI among patients younger than 45 years of age [132]. Conversely, Gonzalez-Heredia et al. found no significant effect of age on weight loss following bariatric surgery [133].

Concerning the influence of age on pharmacotherapy, a substantial portion of data, particularly for GLP-1RAs, stems from post hoc analyses of RCTs. The analysis of the SUSTAIN 1-5 studies with semaglutide, which included a total of 3045 patients, serves as an example, showing no evidence that younger age (<65 years) or older age (≥65 years) influenced the degree of body weight reduction [134]. Consistent findings were observed in the results of the data analyses pertaining to the SUSTAIN 6 and PIONEER 6 trials [135] and also to the PIONEER 9 and 10 studies, concerning the Japanese population [136]. Consistent with experimental data that indicate no significant effect of age on the pharmacokinetics of liraglutide [137], analysis from the phase III studies also showed no influence of age group (<65 years vs. ≥65 years) on body weight loss [138]. Isolated reports from tirzepatide studies in Asian populations indicate that younger patients (<65 years) may experience enhanced benefits from the treatment, possibly due to a lower incidence of AEs [139,140]. Further research into the impact of age on the effectiveness of specific treatments may help determine the most optimal therapeutic option for a given patient. Furthermore, life expectancy, which is partially related to age, should also be considered when selecting a treatment to ensure that the benefits of the treatment outweigh the potential risks.

#### 3.7.2. Sex

Sex is a recognized factor that can influence the development of obesity, its significance, and effectiveness of its therapy. Significant roles in the success of anti-obesity treatment are played by both its biological aspects, which manifest themselves, among other things, in the concentration of sex hormones and differences at the genetic level, as well as the psychosocial aspects, which may be reflected in the perception of excess body weight and different motivations for undertaking its treatment [141]. A significant limitation that hinders the full assessment of the impact of sex on the effectiveness of obesity treatment is the marked overrepresentation of women in clinical trials on obesity. According to the work of Pagoto et al., women constitute, on average, 73% of study participants, despite the fact that obesity is diagnosed in 32.2% of men and 35.5% of women. The authors suggest that this phenomenon may stem from the difference in the perception of obesity between men and women, as well as from women seeking medical advice in this area more frequently [142].

Williams et al., in a systematic review and meta-analysis, assessed 58 RCTs for the effect of sex on weight loss after different non-pharmacological interventions. In terms of absolute weight loss, the reduction was statistically greater in men within the group utilizing dietary interventions alone, and in the group using a combination of dietary intervention and increased physical activity. Conversely, when the percentage of body weight loss was assessed, it was statistically higher in men only in the group that used the combination of dietary intervention and increased physical activity. Regarding the reduction in BMI, it was statistically greater in men utilizing dietary interventions alone. However, in the groups employing increased physical activity or a combination of the aforementioned methods, no statistically significant difference in the magnitude of BMI reduction was observed between the sexes [143].

Risi et al. analyzed the effect of sex on the response to surgery in a meta-analysis concerning surgical treatment. They concluded that men achieved a greater reduction in BMI. Conversely, women were significantly more likely to be classified as therapy responders (2.97; 95% CI: 1.90–4.34) and also showed a greater percentage loss of excess weight [144]. The authors pointed out that the results may be attributable to different perceptions of excess body weight between the sexes. They also noted the significant predominance of women among individuals undergoing bariatric surgery (80.7%) [144,145].

Regarding pharmacological treatment, it is worth mentioning a meta-analysis of 14 studies that utilized various GLP-1RAs. The analysis concluded that greater weight loss was observed in women (mean difference [MD] 1.04 kg [95% CI: 0.70–1.38]) [146]. This discrepancy may be attributed to differences in the pharmacokinetics of incretin mimetics between the sexes. For instance, women exhibited a 32% higher exposure to liraglutide compared to men of equivalent body weight [147,148]. However, concerning tirzepatide, the SURMOUNT-1 trial demonstrated no significant effect of sex on the magnitude of body weight lost [149]. Moreover, a higher percentage of weight loss was also observed in women following the administration of sibutramine [150,151].

Due to the fact that sex is a non-modifiable factor and due to the differences in the perception of obesity by different sex, it seems that an active search for obesity, especially in men, and open communication focused on informing about the impact of obesity on health and current methods of its treatment may be important in improving the results of obesity treatment.

#### 3.7.3. Pretreatment BMI

BMI remains one of the most popular and simplest indicators utilized for initial nutritional screening in patients. However, due to its inherent limitations, such as the observation that high values do not always reflect excessive adipose tissue accumulation, it is considered insufficient for a comprehensive assessment of patients with obesity [152]. Another caveat regarding the reliance on BMI as a predictor of anti-obesity therapy success is the potential discrepancy between the BMI distribution observed in research cohorts and that of the broader population. Specifically, concerns exist about the insufficient inclusion of subjects possessing the most extreme BMI values within the qualified study sample [153]. Despite these imperfections, the response of patients to treatment for excess body weight was assessed depending on their initial BMI in many studies.

In a study conducted by Stubbs et al., the impact of baseline BMI on absolute and relative body weight loss was assessed. The researchers analyzed data derived from 34,271 patients who participated in a commercial, 12-week program aimed at promoting weight reduction through lifestyle education, including dietary counselling. Participants were stratified into four distinct groups based on their baseline BMI. It was found that the higher the BMI group, the greater the absolute weight loss; conversely, the percentage of body weight loss was comparable across all groups. The limitations of this study include the lack of a control group and a limited follow-up of only 12 weeks [154].

In the context of surgical interventions, the study by Mantziari et al. is notable, as it assessed the influence of baseline BMI on the outcomes following RYGB surgery. For this investigation, 957 patients were stratified into two cohorts: superobese (BMI > 50 kg/m^2^) and morbidly obese (BMI 35–50 kg/m^2^). The cohort with the higher baseline BMI retained a significantly higher post-operative BMI compared to the other group (mean BMI 39.1 kg/m^2^ vs. 30.8 kg/m^2^, *p* < 0.001), yet they exhibited a similar relative weight loss (28.3% vs. 28.8%, *p* = 0.644). Crucially, remission of T2DM occurred with a comparable frequency in both cohorts (39.0% vs. 40.9%, *p* = 0.335) [155]. Similarly, a retrospective study on SG, which compiled data from 540 patients across 17 Spanish centers, yielded comparable results: it was observed that patients with a higher initial BMI experienced a smaller percentage reduction in excess body weight post-procedure [156]. Furthermore, two separate investigations into bariatric surgical outcomes noted a comparable trend: a higher baseline BMI was associated with a smaller decrease in the percentage of excess body weight [157,158].

Regarding treatment with GLP-1RAs, a notable finding is presented in a meta-analysis of 55 RCTs. This comprehensive study, which assessed 12 distinct GLP-1RAs and included 16,269 patients, did not demonstrate a significant influence of baseline BMI on the magnitude of weight loss achieved [159]. The impact of BMI on the efficacy of semaglutide pharmacotherapy was evaluated in another subgroup meta-analysis of RCTs. This analysis synthesized data from 13 RCTs, encompassing 5838 participants, of whom 3794 received subcutaneous semaglutide. Participants were stratified into two groups based on baseline BMI. The findings indicated that patients with higher baseline BMI achieved a greater absolute weight loss, whereas those in the lower BMI group demonstrated a higher proportion of individuals attaining a 5% and 10% reduction from their baseline body weight [160]. A parallel subgroup analysis of the semaglutide SUSTAIN 1-5 RCTs, wherein patients were categorized into four groups based on baseline BMI, revealed that patients with higher pre-treatment BMI had greater absolute weight loss. However, this specific study did not detect a significant influence of baseline BMI on relative weight loss [161]. Another relevant investigation is a systematic review and meta-analysis of 56 RCTs, comprising a large cohort of 60,307 patients (32,598 of whom were in active treatment groups). This study evaluated the influence of various factors on the efficacy of pharmacological anti-obesity treatments, encompassing GLP-1RAs and agents such as orlistat, naltrexone-bupropion combination, and phentermine-topiramate combination. While the authors presented the absolute weight loss stratified across four baseline BMI groups for each medication, they simultaneously noted insufficient available data, particularly within the extreme BMI categories (overweight and BMI ≥ 40 kg/m^2^) [162]. It is also worth mentioning a study that analyzed the impact of many clinical variables on the outcomes of pharmacological treatment for obesity in the pediatric population (children and adolescents). Overall, this investigation reported greater absolute weight loss in patients who presented with a higher BMI [163].

BMI is an important factor influencing the prognosis and the choice of therapeutic method, and further research into its impact on the effectiveness of obesity treatment may help to better utilize it, but it should not be interpreted in isolation from the remaining clinical context.

#### 3.7.4. Obesity Phenotypes and Body Composition

Given the limitations of BMI in assessing excess adiposity and predicting its complications, classifying individuals into subgroups offers crucial supplementary information for patient risk assessment. This division distinguishes four principal phenotypes: metabolically healthy obese (MHO), metabolically unhealthy obese (MUO) (obese BMI with significant metabolic dysfunction), metabolically obese with normal weight (MONW) (normal BMI, yet exhibiting metabolic abnormalities typical of obesity) and sarcopenic obesity (SO) (concurrent excess adiposity and reduced muscle mass) [164,165]. An alternative approach to assessing body composition in individuals with obesity involves evaluating the content of individual types of adipose tissue, with a particular emphasis on subcutaneous adipose tissue (SAT) and visceral adipose tissue (VAT). Of these, higher amounts of VAT are recognized as being most highly correlated with metabolic complications [166]. Numerous methodologies are available for assessing VAT in patients, including dual X-ray absorptiometry, ultrasound, computed tomography (CT), and nuclear magnetic resonance imaging (NMRI) or magnetic resonance imaging (MRI) [167].

Distinct weight loss modalities exert varying effects on body composition; therefore, precise baseline assessments of a patient’s body composition may facilitate the selection of an optimal therapeutic intervention. For instance, increased physical activity promotes adiposity reduction while preserving lean body mass [168,169,170]. On the other hand, clinical data regarding tirzepatide pharmacotherapy indicate that the composition of weight loss remains consistent regardless of total magnitude; specifically, the ratio of fat mass reduction to lean body mass loss maintains a proportional threshold of approximately 3:1 [149]. Moreover, findings from the Chaston and Dickson systematic review reveal a non-linear relationship between weight reduction and adipose tissue distribution. Specifically, initial modest weight loss is characterized by the preferential mobilization of visceral adipose tissue; however, this targeted effect appears to attenuate as the magnitude of total weight loss increases [171].

Another parameter used to assess the body composition of individuals with obesity is muscle mass or muscle strength, expressed, for example, by handgrip strength. Obese patients with low muscle mass or strength are classified as sarcopenic obese. This phenotype of patients is important because it has been shown that patients with sarcopenia and sarcopenic obesity are characterized by increased mortality compared to the rest of the population [172,173,174]. In this case, it was assessed that bariatric treatment is equally safe and effective in the context of remission of concomitant diseases and that metabolic changes after surgery are similar in groups with and without sarcopenia [175,176]. Further research in this area, especially the evaluation of specific treatment methods, is necessary and may help to select the appropriate therapy for a given patient and thus improve the effectiveness of treatment.

Given the significant phenotypic heterogeneity in body composition among individuals with obesity, further empirical investigation is warranted to facilitate a precision medicine approach. Such research may enable the optimization of therapeutic selection tailored to the specific physiological profile of each patient.

#### 3.7.5. Microbiome

There is scientific data indicating that in many diseases, including metabolic diseases such as obesity or T2DM, the composition of the gastrointestinal microflora may differ from that of healthy people [177,178,179]. The intestinal microbiota modulates host metabolism through several key mechanisms, most notably by facilitating macronutrient digestion, regulating bile acid metabolism, and synthesizing short-chain fatty acids (SCFAs). Furthermore, these microbial communities influence metabolic homeostasis by modulating the secretion of pro-inflammatory cytokines, metabolic hormones, and neurotransmitters. A widely utilized metric for characterizing dysbiosis is the *Firmicutes*-to-*Bacteroidetes* (F/B) ratio. Extensive literature suggests that an elevated F/B ratio is positively correlated with obesity, reflecting a microbial profile optimized for increased energy harvest from the diet [180]. Moreover, it has also been shown that some drugs used to treat obesity may affect the composition of the gastrointestinal microflora for example orlistat or GLP-1RAs [181,182,183].

The importance of gastrointestinal microbiota in weight loss was demonstrated by Pereira et al. in a study comparing the effectiveness of a multimodal intervention designed to support a change in the composition of the gut microbiota with a hypocaloric diet. The authors concluded that the abundance of *Faecalibacterium* in the microbiota was associated with greater fat loss in the group undergoing the complex behavioral intervention [184]. Another study found that a higher ratio of *Prevotella* to *Bacteroidetes* was associated with greater body fat loss in participants consuming a high-fiber, whole-grain diet [185].

In the domain of bariatric surgery, the seminal research conducted by Stefur et al. provides critical insights into how baseline microbiota composition influences the efficacy of RYGB. The investigators demonstrated that patients achieving optimal surgical outcomes were characterized by a higher relative abundance of the phylum *Firmicutes* within the oral cavity and the genus *Tannerella* (phylum *Bacteroidetes*) within the intestinal flora. Conversely, patients with a suboptimal therapeutic response exhibited a gut microbiome enriched with *Deltaproteobacteria* (phylum *Proteobacteria*) and the family *Barnesiellaceae* (phylum *Bacteroidetes*). These results should be interpreted cautiously due to the pilot nature of the study and the very small study group [186].

Further research in this area may provide interesting data to improve the effectiveness of obesity treatment.

#### 3.7.6. Sleep Duration and Quality

The length and quality of sleep is another factor that may influence the effectiveness of obesity treatment, but the interactions between the length and quality of sleep and excess weight may be bidirectional [187]. For example, in a study involving 245 obese women who underwent an intervention consisting of dietary modification and physical activity advice, the impact of sleep quality assessed using the Pittsburgh Sleep Quality Index (PSQI) Global Score or sleep duration (<7 h vs. >7 h per night) was assessed. It was found that better sleep quality or duration increased the likelihood of therapeutic success in non-pharmacological treatment of obesity. However, since the study included only women and relied on subjective assessments of sleep quality, these results should be interpreted with caution [188]. Moreover, the study by Bogh et al. found that better sleep quality or duration helped maintain the effects of weight loss treatment [189]. Moreover, emerging evidence suggests that suboptimal sleep quality may attenuate the efficacy of bariatric interventions, potentially leading to diminished weight loss outcomes. However, the relatively small sample size highlights the need for further research in this area [190,191]. Furthermore, in a Japanese cohort of individuals with obesity undergoing oral semaglutide therapy, prolonged sleep duration was positively correlated with enhanced therapeutic outcomes and more pronounced weight reduction. The subjective nature and self-reported assessment of sleep quality are limitations of this study and indicate the need for further research in this field [192]. Therefore, it is worth actively looking for sleep disorders in patients with obesity and referring them to centers that can provide comprehensive treatment.

### 3.8. Polypharmacy

Obesity is frequently characterized by various comorbidities, which may either be pathogenetically linked to the condition as secondary complications or exist as independent, unrelated pathologies [193,194]. A cross-sectional study conducted in England involving 7730 participants found that overweight and obesity were strong risk factors for polypharmacy (defined as the combined use of 5–9 medications) and hyperpharmacy (the combined use of more than 10 medications). For obesity, the hazard ratio for polypharmacy was 1.81 (95% CI: 1.53–2.15, *p* < 0.01), and for hyperpharmacy was 2.28 (95% CI 1.63–3.21, *p* < 0.01) [195]. Many commonly used medications can influence body weight, either promoting gain or loss. Agents known to contribute to weight gain include certain antidiabetics, glucocorticoids, some antipsychotics and antidepressants, as well as specific antiepileptics [196]. The concurrent use of weight-promoting medications for comorbidities may reduce the effectiveness of obesity interventions, potentially resulting in pseudo-resistant or pseudo-refractory obesity. Notably, the underlying pathophysiological mechanisms of drug-induced weight gain vary significantly across different therapeutic classes. However, a detailed discussion of this topic is beyond the scope of this paper, and therefore, only selected issues will be mentioned below.

Patients with obesity should be assessed for comorbidities and all current medications, with particular attention to drugs that may promote weight gain. If such drugs are identified, clinicians should consider switching to alternatives with a more favorable metabolic profile or discontinuing the medication when feasible.

#### 3.8.1. Antidiabetic Medications

Due to the frequent co-occurrence of T2DM and obesity, the effect of insulin on body weight seems to be non-negligible. The weight-promoting effects of insulin therapy are multifaceted, involving both metabolic and behavioral pathways. Metabolically, insulin facilitates energy storage through the upregulation of lipogenesis and the suppression of lipid mobilization. Clinically, the resolution of glycosuria leads to the retention of calories previously excreted in urine. These physiological shifts are frequently compounded by behavioral compensations, specifically increased caloric intake driven by the fear of hypoglycemia [197].

Regarding oral antidiabetic agents, the weight gain observed with sulfonylureas is primarily driven by stimulated insulin secretion that occurs independently of ambient blood glucose concentrations. Conversely, the weight gain associated with thiazolidinediones is thought to stem from a distinct mechanism involving the systemic redistribution of adipose tissue; specifically, these agents promote the expansion of subcutaneous fat depots while concurrently reducing visceral adiposity [198,199].

#### 3.8.2. Glucocorticosteroids

In the case of glucocorticosteroids, which are known to increase the body weight of individuals treated with them, the mechanisms behind the increase in body weight are also complex and result from both the effect on the central regulation of appetite and the effect on peripheral adipose tissue [200].

#### 3.8.3. Psychotropic Medications

Among antipsychotic medications, olanzapine, quetiapine, and risperidone can lead to weight gain. Of these medications, olanzapine causes the greatest increase in body weight [196]. This is believed to be due to a central increase in appetite, and therefore, increased calorie intake [201].

In the case of antidepressants, popular compounds with proven weight gain include amitriptyline and mirtazapine [196]. In the case of mirtazapine, it has been shown to increase appetite, particularly for sweet foods [202].

#### 3.8.4. Antiepileptic Medications

Another group of drugs containing agents capable of increasing the body weight of treated individuals is antiepileptic drugs, and specific substances with this effect include valproic acid/sodium valproate, carbamazepine and gabapentin [196]. Also in the case of these drugs, the mechanisms leading to weight gain in carbamazepine or valproate recipients are complex and not fully understood, but they result from the influence on both the central control of satiety and the differentiation of adipose tissue [203,204]. In contrast, in terms of gabapentin intake, weight gain is assigned to alterations in function of the gastrointestinal system and to fluid retention related to increased permeability of peripheral vessels [205].

### 3.9. Selected Comorbidities Associated with an Increased Risk of Obesity

Several hormonal disorders are known to contribute to weight gain by altering metabolic rate, appetite regulation, body storage and utilization of energy. Among the endocrine conditions most commonly associated with an increased risk of obesity are PCOS, hypothyroidism, hyperadrenocorticism, hyperinsulinemia or growth hormone deficiency. These conditions should be ruled out before diagnosing resistant or refractory obesity.

#### 3.9.1. PCOS

PCOS is a common condition affecting women of reproductive age; it is characterized by ovulation and fertility disorders, as well as metabolic abnormalities. The pathogenesis of the disease is considered multifactorial, involving key drivers such as hyperandrogenism, neuroendocrine disruption, hypothalamic-pituitary-ovarian (HPO) axis dysfunction, and insulin resistance [206]. The condition can manifest in young, lean women without other metabolic disorders; however, estimates suggest that obesity, particularly visceral obesity, affects roughly 50% of the PCOS population [207,208]. The underlying disorders of PCOS and obesity exacerbate one another in a vicious circle. By heightening insulin resistance and inflammatory adipokine production, obesity triggers hyperinsulinemia, leading to stimulated adipogenesis and suppressed lipolysis. Obesity sensitizes theca cells to luteinizing hormone (LH) stimulation, leading to increased ovarian androgen secretion. In turn, testosterone drives the accumulation of visceral fat, creating a feedback loop that further worsens insulin resistance [208,209]. Difficulties in obesity treatment in patients with PCOS may also stem from reward system dysfunction and delayed diagnosis of eating disorders such as EE, BED, and NES. According to Marsh et al., patients with PCOS and insulin resistance exhibit increased limbic system activity during emotion-related tasks compared to the control group. This pattern was not observed after initiating metformin therapy [90,210]. As with other patients with excess body weight, first-line treatment should be based on lifestyle and dietary changes, the introduction of exercise or behavioral therapy. When these treatments are insufficient, pharmacological methods should be used, the most common of which is metformin. Metformin improves insulin sensitivity, lowers androgen levels, and promotes weight loss, including waist circumference, in combined therapy with GLP-1RAs shows particular effectiveness [206].

#### 3.9.2. Hypothyroidism

Thyroid hormones play an important role in regulating the body’s metabolic homeostasis, both by influencing basal metabolism rate and by increasing resting energy expenditure. Weight gain or difficulty losing weight may be the first symptom noticed by these patients, prompting them to seek medical attention. It results from water retention, increased glycosaminoglycan accumulation, and an expansion of adipose tissue. Research demonstrates a linear correlation between thyroid-stimulating hormone (TSH) and BMI, where elevated TSH levels are associated with an increased propensity for weight gain [211,212]. Available data indicate that despite achieving euthyroidism, 82% of women with a history of Hashimoto’s disease—the most common cause of hypothyroidism—are overweight and 35% are obese [213,214]. Nonetheless, non-pharmacological strategies centered on caloric deficit, alongside contemporary anti-obesity pharmacotherapy, seem highly effective within this patient group. In a six-month study by Ostrowska et al., which included 100 women with Hashimoto’s disease, participants were divided into two groups: one followed a standard reduction diet, while the other eliminated food allergens. A significant decrease in BMI and body fat levels was observed in both groups, although the reduction was significantly greater in the former (*p* < 0.002 and *p* = 0.026, respectively) [213]. According to a PLoS One study published in September 2025, weight loss outcomes were comparable between 53 patients with hypothyroidism on stable levothyroxine and 145 age-, gender-, and BMI-matched controls. After a 10-month median follow-up on 3 mg liraglutide, no significant differences were found in weight loss (−10.8% vs. −8.9%, *p* = 0.940) or lean body mass reduction (−29.9% vs. −33.3%, *p* = 0.729) [215]. Bariatric surgery has also been shown to be equally effective. A retrospective cohort study compared total weight loss one year after RYGB or SG in patients taking levothyroxine for hypothyroidism versus those without the condition; the results were 28.0% ± 9.9 and 26.7% ± 9.5 (*p* = 0.46), respectively [22]. However, a certain limitation may be a slightly increased risk of long-term complications of the above-mentioned treatments, such as deficiencies of B vitamins, iron or the risk of developing osteoporosis, incident cerebrovascular events, T2DM, dyslipidemia, hypoglycemia or polyneuropathy in patients with hypothyroidism [216].

C. Issues with maintaining weight loss

The treatment of obesity may be generally divided into two phases—weight reduction and the following focus of keeping proper body weight. The latter is especially problematic after cessation of therapy. Weight regain may have many reasons; the majority are unaddressed factors that have been described above. However, there are also factors connected with altered metabolism.

### 3.10. Weight Regain After Bariatric Surgery

Long-term weight maintenance after bariatric surgery remains a major clinical challenge (Table 3). Although surgical interventions generally achieve superior outcomes compared with non-surgical therapies, a substantial proportion of patients experience suboptimal weight loss or clinically significant weight regain. Suboptimal weight loss—defined as failure to lose 40–60% of excess body weight within two years—occurs in approximately 11–22% of cases. However, weight regain after an initial satisfactory response is more prevalent and constitutes the primary focus of this review [217,218]. The etiology of this process is multifactorial, encompassing psychological factors—such as lack of support, depressive and anxiety disorders, EE or BED, dietary errors, and lack of physical activity—as well as surgical factors, like stretching of the gastric sleeve or pouch which leads to the loss of restriction and reduced satiety. It is also estimated that numerous post-operative changes occur within patients’ bodies, including alterations in biochemical processes, hormonal secretion, and even the composition of the gut microbiota [219]. An interesting observation noted in several studies is the alteration in taste preferences among post-bariatric patients, particularly a shift away from sweet dishes. It applies especially to those patients who have undergone RYGB [220,221]. Hormonal changes concern the secretion of GLP-1, leptin and ghrelin, which are involved in regulating the feeling of hunger and satiety, as well as the body’s energy expenditure. Based on available meta-analyses, a trend toward increased postprandial GLP-1 secretion has been observed in patients following RYGB or SG, while fasting GLP-1 levels remain largely unchanged. This enhanced secretion seems to be associated with successful weight loss after bariatric surgery [222]. In the study by le Roux et al., patients with insufficient weight loss following gastric bypass demonstrated significantly lower secretion of both peptide YY and GLP-1 compared to those who achieved successful weight loss (*p* < 0.05) [223]. Similarly, in a cross-sectional study of 34 patients, Shantavasinkul et al. demonstrated that individuals with sustained weight loss five years after RYGB exhibited significantly higher postprandial GLP-1 levels compared to those experiencing weight regain, despite similar fasting GLP-1 concentrations [224]. Given regularities observed, scientists investigated whether GLP-1RAs could be used in the pharmacotherapy of this patient group. For example, in the BARI-OPTIMISE double-blind, placebo-controlled trial, 70 patients who underwent bariatric intervention and exhibited an insufficient postprandial GLP-1 response were randomized to receive either liraglutide (3 mg/day) or a placebo, alongside lifestyle interventions. By week 24, the liraglutide group showed an 8.82% reduction in body weight, while the placebo group remained stable. Furthermore, 71.9% of those treated with liraglutide achieved a weight loss of over 5%, compared to only 8.8% in the placebo group. The estimated treatment difference of −8.03% was notably higher than the −4.8% difference typically observed in non-bariatric patients, as reported in a recent meta-analysis of RCTs [222,225].

### 3.11. Weight Regain After Cessation of Pharmacotherapy

The cessation of anti-obesity pharmacotherapy is almost invariably followed by a rapid and significant regain of body weight, a phenomenon now understood not as a failure of patient willpower, but as a predictable physiological relapse characteristic of chronic obesity.

The Clinical Evidence Recent discontinuation trials have quantified this trajectory with stark precision. In the STEP 1 Extension study, participants who withdrew from semaglutide treatment after 68 weeks regained approximately two-thirds of their lost weight within the subsequent year [226]. Similarly, the SURMOUNT-4 trial demonstrated that patients treated with tirzepatide for 36 weeks who were then switched to placebo experienced a substantial 14% weight regain over the following year, while those who remained on therapy continued to lose weight. Crucially, the cardiometabolic improvements achieved during treatment—such as reductions in blood pressure, lipids, and HbA1c—reverted toward baseline in lockstep with the weight recurrence [227].

### 3.12. Metabolic Rebound

This rapid “rebound” is driven by a potent biological counter-regulation known as the “energy gap”. During active weight loss, the body undergoes metabolic adaptation: resting energy expenditure drops disproportionately to the loss of tissue, while appetite hormones (like ghrelin) increase to drive food seeking [231]. AOMs function by artificially suppressing this heightened drive and promoting satiety.

When the medication is withdrawn, this suppression is abruptly lifted. The patient is left with a suppressed metabolic rate but an unmasked, intense biological drive to eat. Furthermore, the body enters a state of hyper-efficient lipogenesis, where it prioritizes the rapid replenishment of energy stores. Preclinical models suggest that during this early relapse phase, calories are shunted into fat storage with near-perfect efficiency (~98%), often leading to a preferential regain of fat mass over lean muscle [232].

The solution for weight loss after cessation of treatment might be a gradual supervised reduction in drug dosing and long-term follow-up with early interventions during weight gain.

## 4. Proposed Clinical Workflow

Based on this review, we propose the following clinical workflow to improve outcomes. This workflow integrates biological, psychiatric, and socioeconomic determinants to optimize “phenotype-guided” treatment strategies:

Step 1: Comprehensive Baseline Assessment
  - Anthropometrics: Calculate BMI and assess body composition (visceral vs. subcutaneous adipose tissue). Evaluate muscle strength.
    ○ BMI alone may miss “Metabolically obese normal weight” (MONW) or sarcopenic obesity phenotypes.
  - Medication review: Screen for “weight-promoting” drugs (e.g., antipsychotics like olanzapine, antidepressants like mirtazapine, insulin, sulfonylureas, glucocorticoids).
    ○ Consider deprescribing or switching to weight-neutral alternatives if clinically feasible.
Step 2: Psychiatric and behavioral screening
  - Screen for depression: Use tools like the Beck Depression Inventory.
    ○ If depression is present, treat it. Untreated depression compromises weight loss.
    ○ Drug choice: bupropion + escitalopram is superior for combined obesity/depression. Fluoxetine may also help. Use GLP-1RAs with caution and monitor for mood changes.
  - Screen for anxiety: assess for persistent worry/restlessness.
    ○ Drug choice: avoid phentermine/topiramate or naltrexone/bupropion as they can exacerbate anxiety. Prefer GLP-1RAs or tirzepatide, which are not linked to increased anxiety risk.
  - Screen for eating disorders: Check for binge eating disorder (BED), emotional eating (EE), or night eating syndrome (NES).
    ○ Drug choice: lisdexamfetamine is approved for BED. naltrexone/bupropion or GLP-1RAs can target reward-driven/emotional eating. Avoid orlistat (ineffective for BED).
Step 3: Phenotype-based drug selection
  - Match the medication mechanism to the patient’s dominant obesity phenotype (Table 4).
Step 4: Socioeconomic and practical assessment
  - Cost and access: verify insurance coverage. Newer incretins (semaglutide/tirzepatide) are costly; older agents (phentermine/topiramate) may be necessary if finances are a barrier.
  - Route of administration: assess willingness to inject.
    ○ Needle anxiety or cognitive impairment in elderly may necessitate oral options (e.g., oral semaglutide, naltrexone/bupropion).
Step 5: Monitoring
  - The 3–6 month rule: assess weight loss at 3–6 months.
    ○ Definition of Responder: at least 5% weight loss (3% for diabetics).
    ○ If threshold is not met, discontinue and switch therapy.
Step 6: Pharmacogenomic profiling (near future)
  ○ Monogenic Obesity (*MC4R*, *POMC*, *LEPR*, *PCSK1*)
  ○ GLP-1 Resistance
Step 7: Drug discontinuation or maintenance therapy
  ○ Slow dose deescalation
  ○ Surveillance and early reinstatement of pharmacotherapy
  ○ Optional long-term low dose therapy

## 5. Conclusions

The paper identifies and synthesizes the primary determinants influencing the efficacy of obesity treatment. Given the profound implications of successful weight management for individual health, public wellness, and the sustainability of healthcare systems, the early identification of barriers to treatment success is paramount. Such proactive screening facilitates timely clinical intervention for modifiable risk factors. Conversely, for non-modifiable factors, early identification ensures the prompt referral of high-risk patients to specialized bariatric or metabolic centers, thereby optimizing long-term clinical outcomes.

**Figure 1:**
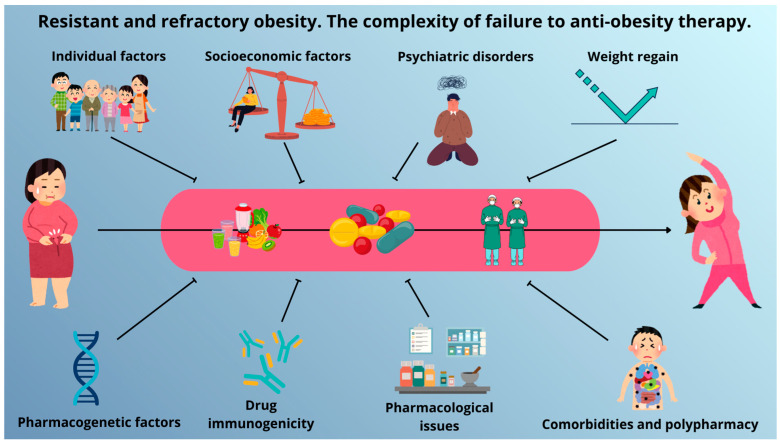
Graphical summary of the determinants of obesity treatment effectiveness.

The preceding sections provide a comprehensive analysis of the multifactorial determinants influencing the efficacy of obesity treatment, which are summarized in Figure 1. This review evaluates the role of both physical and psychiatric comorbidities, specifically examining how the concurrent management of these conditions modulates weight-loss outcomes. Additionally, the analysis addresses the significant impact of socioeconomic determinants, which exert a non-negligible influence on therapeutic success. Furthermore, individual patient variables are scrutinized in the context of their interaction with specific therapeutic modalities. The discussion concludes with a synthesis of factors that may disrupt the optimal pharmacokinetics of pharmacological agents utilized in obesity management.

This review underscores the inherent complexity of obesity and the nuances involved in achieving therapeutic efficacy. To improve the identification of patients predisposed to suboptimal treatment responses, further longitudinal research is essential. Specifically, the development of robust predictive models to forecast individual treatment trajectories represents a critical frontier in personalized obesity management. Given the multifaceted nature of the disease, a shift from solitary clinical practice toward a holistic, interdisciplinary and multidisciplinary team-based approach is essential for successful intervention. Finally, patients identified with a high baseline risk of treatment resistance, or those exhibiting inadequate responses to conventional therapies, should be prioritized for referral to specialized tertiary centers, where state-of-the-art pharmacotherapy can be provided alongside comprehensive behavioral, lifestyle, and surgical interventions.

## Figures and Tables

**Table 1:** Mechanisms of action, common AEs, overall efficacy, and rates of treatment failure and discontinuation of currently approved obesity therapies.

Intervention	Mechanism of Action	Common Drug-Related AEs	Peak Weight Loss (% Total Body Weight)	Study Context	Non-Response Rate /Pharmacotherapy Discontinuation Rate	Definition of Non-Responder	Reference
Bariatric Surgery							
Roux-en-Y gastric bypass (RYGB)	Surgery (malabsorptive)	Not Applicable	~25–30%	Considered the “gold standard” for severe obesity. Effects are typically sustained long-term (≥10 years).	~8.5%/Not Applicable	Failure to achieve >50% excess weight loss at the 2-year follow-up.	[7,21,22,23]
Sleeve Gastrectomy (SG)	Surgery (restrictive)	Not Applicable	~20–25%	Less invasive than RYGB with comparable short-term results, though slightly lower long-term weight maintenance.	~23%/Not Applicable	Failure to achieve >50% excess weight loss at the 2-year follow-up.	[7,21,22,24]
Pharmacotherapy							
Retatrutide (Investigational)	Triple Glucagon/GIP/GLP-1RA	Nausea, diarrhea, vomiting, constipation	~24% (Preliminary mean body-weight loss compared with 2.1% weight loss in the placebo group)	Phase II data: 338 adults (52% men) with obesity or overweight (BMI ≥ 27 kg/m^2^ and ≥1 weight-related coexisting condition) were randomly assigned in a 2:1:1:1:1:2:2 ratio to receive subcutaneous retatrutide (1 mg, 4 mg [initial dose, 2 mg], 4 mg [initial dose, 4 mg], 8 mg [initial dose, 2 mg], 8 mg [initial dose, 4 mg], or 12 mg [initial dose, 2 mg]) or placebo once weekly for 48 weeks. Age: 48.2 ± 12.7 yr Baseline BMI: 37.3 ± 5.7 kg/m^2^ Mean body-weight loss observed in the 12 mg dose group after 48 weeks of once-weekly injections exceeded the weight loss reported with tirzepatide	0% / 6% in the 8 mg dose group and 16% in the 12 mg dose group (Preliminary)	Phase II data showed 100% of patients in both the 8 mg and 12 mg dose groups achieved ≥5% weight loss at 48 weeks	[20]
Tirzepatide	Dual GIP/GLP-1RA	Nausea, diarrhea, vomiting, constipation	~20% (compared with 13.7% weight loss in the semaglutide group; *p* < 0.001)	SURMOUNT-5 Trial (phase IIIb) data: 750 adults (35.3% men) with obesity but without T2DM were randomly assigned in a 1:1 ratio to receive the maximum tolerated dose of tirzepatide (10 mg or 15 mg) or the maximum tolerated dose of semaglutide (1.7 mg or 2.4 mg) subcutaneously once weekly for 72 weeks. Age: 44.70 ± 12.84 yr Baseline BMI: 39.5 ± 7.6 kg/m^2^ The only approved agent currently demonstrating efficacy approaching that of bariatric surgery.	~9%/6.1%	Failure to achieve ≥5% total weight loss.	[3,6,7]
Semaglutide (2.4 mg)	GLP-1RA	Nausea, vomiting, constipation, diarrhea, abdominal pain	~14% (compared with 2.4% weight loss in the placebo group; *p* < 0.001)	STEP Trials data: 1961 adults (25.9% men) with obesity or overweight (BMI ≥ 27 kg/m^2^ and ≥1 weight-related coexisting condition except T2DM) were randomly assigned in a 2:1 ratio to 68 weeks of treatment (approx. 18 months) with once-weekly subcutaneous semaglutide (at a dose of 2.4 mg) or placebo, plus lifestyle intervention Age: 46 ± 13 yr Baseline BMI: 37.9 ± 6.7 kg/m^2^	13.6%/7%	Failure to achieve ≥5% total weight loss.	[6,16,25]
Orlistat	Lipase inhibitor	Oily/fatty feces, bloating, fecal urgency	~5–10% compared with 2.8% weight loss in the placebo group (*p* < 0.001)	XENDOS Trial data: 3305 adults (44.4% men) with obesity and without T2DM were randomly assigned to lifestyle changes plus either oral orlistat 120 mg or placebo, three times daily in a 1:1 ratio for 4 years Age: 43 ± 8 yr Baseline BMI: 37.3 ± 4.2 kg/m^2^	~43%/48%	Failure to achieve ≥5% total weight loss.	[11,18,26]
Phentermine/topiramate	Sympathomimetic/GABA modulation	Dizziness, insomnia, constipation	9.8% (at the highest dose of 15/92 mg compared with 1.2% weight loss in the placebo group; *p* < 0.0001)	CONQUER Trial data: 2487 adults (30.2% men) with obesity or overweight (BMI ≥ 27 kg/m^2^ and ≥2 comorbidities such as hypertension, dyslipidaemia, diabetes or prediabetes, or abdominal obesity) were randomly assigned to placebo, once-daily oral phentermine 7.5 mg plus topiramate 46 mg, or once-daily oral phentermine 15 mg plus topiramate 92 mg in a 2:1:2 ratio for 56 weeks Age: 51.1 ± 10.4 yr Baseline BMI: 36.6 ± 4.5 kg/m^2^	30%/ 31% in the 7.5/46 mg dose group and 36% in the 15/92 mg dose group	Failure to achieve ≥5% total weight loss.	[9,17]
Liraglutide (3.0 mg)	GLP-1RA	Nausea, vomiting, constipation, diarrhea, abdominal pain	~8% (compared with 2.6% weight loss in the placebo group; *p* < 0.001)	SCALE Trials data: 3731 adults (21.5% men) with obesity or overweight (BMI ≥ 27 kg/m^2^ with treated or untreated dyslipidemia or hypertension) but without T2DM were randomly assigned in a 2:1 ratio to 56 weeks of treatment with once-daily subcutaneous liraglutide (at a dose of 3.0 mg) or placebo, plus counseling on lifestyle modification Age: 45.1 ± 12 yr Baseline BMI: 38.3 ± 6.4 kg/m^2^	36.8%/20–28%	Failure to achieve ≥5% total weight loss.	[8,27]
Naltrexone/bupropion	Opioid antagonist/ dopamine reuptake inhibitor	Nausea, vomiting, constipation, diarrhea, dizziness	~6.5% (compared with 1.2% weight loss in the placebo group; *p* < 0.001)	COR-II Trial data: 1496 adults (15.4% men) with obesity or overweight (BMI ≥ 27 kg/m^2^ with dyslipidemia and/or hypertension) were randomly assigned in a 2:1 ratio to 56 weeks of treatment with once-daily oral combined naltrexone sustained-release (SR) plus bupropion SR (32/360 mg) or placebo Age: 44.3 ± 11.2 yr Baseline BMI: 36.2 ± 4.5 kg/m^2^	52%/24.3%	Failure to achieve ≥5% total weight loss.	[10,19,28]

**Table 2:** *GLP1R* polymorphisms—impact on body weight and the effects of GLP-1RAs.

*GLP1R* Gene Polymorphism	Clinical Implications	Study Population	Metabolic Impact	GLP-1 Response	References
rs2268641	Association with excessive weight	600 Polish patients with BMI ≥ 25 kg/m^2^	Homozygous genotype TT – significantly lower risk of excessive body weight	Not Available	[30]
rs6923761	(1) Impact on body mass and plasma glucose levels (2) Body weight response to GLP-1RAs	(1) 600 Polish patients with BMI ≥ 25 kg/m^2^ (2) 57 obese women with PCOS	(1) AA carriers—greater risk of excessive body weight (in comparison to GG) and higher glucose concentration (in comparison to AG) (2) The *GLP-1R* rs6923761 polymorphism accounts for variability in weight loss	At least one rs6923761 minor allele—stronger response to liraglutide treatment	[30,33]
rs10305420	Body weight response to GLP-1RAs	57 obese women with PCOS	The *GLP-1R* rs10305420 polymorphism accounts for variability in weight loss	Individuals with at least one minor allele—lower likelihood of liraglutide treatment success compared to wild-type homozygotes	[33]
rs1042044	Impact on risk of various obesity types	252 children aged 6–18 years with obesity	Association of the C allele with the development of metabolically unhealthy obesity (MUO) in children	Not Available	[35]

**Table 3:** Relapse in obesity among various treatment options.

Treatment Modality	Relapse/Regain Rate	Context and Timeframe	Reference
Pharmacotherapy	(Regain upon discontinuation)	–	–
Semaglutide (2.4 mg)	Regained ~67% of lost weight	STEP 1 Extension: Participants regained two-thirds of their prior weight loss 1 year after stopping the medication.	[226]
Tirzepatide	~82% of patients regained weight	SURMOUNT-4: 82% of patients regained at least 25% of their lost weight within 1 year of switching to placebo.	[227]
Liraglutide (3.0 mg)	~19% total relapse	Real-World Data: ~19% of users regained all lost weight (or more) 1 year after stopping; most others regained a significant portion.	[228]
Phentermine/topiramate	Trend to baseline	Prospective Study: 6 months after discontinuation, weight and metabolic parameters showed a clear trend returning to pre-treatment baseline.	[229]
Bariatric Surgery	(Long-term recidivism)	–	–
General surgical outcome	20–25% of patients	Manuscript: “Significant weight gain occurs in as many as 20–25% of patients after bariatric surgery.”	[217]
Sleeve gastrectomy (SG)	~76% of patients	Systematic Review: Up to 76% of patients experienced significant weight regain at 6-year follow-up.	[230]
Gastric bypass (RYGB)	~37% of patients	Long-Term Cohort: Approximately 37% of patients had significant regain (defined as ≥25% increase from lowest weight) at 7-year follow-up.	[230]

**Table 4:** Suggested therapeutic approach based on clinical assessment.

Phenotype	Preferred Mechanism of Drug Action	Drug of Choice	Reference
Impaired satiation/abnormal satiety	Satiety induction	GLP-1RAs (semaglutide, liraglutide) and dual incretin agonist (tirzepatide) target central appetite regulation.	[47,48]
Cravings/reward-driven	Reward pathway modulation	Naltrexone/bupropion targets dopaminergic reward pathways. GLP-1RAs also reduce hedonic drive.	[47,48]
T2DM/ insulin resistance	Glycemic control + weight loss	Tirzepatide or semaglutide are first-line for efficacy.	[5]
PCOS	Insulin sensitization	Metformin + GLP-1RAs combination is particularly effective.	[233,234]

## Data Availability

No new data have been created. Data sharing is not applicable to this article.

## Abbreviations

The following abbreviations are used in this manuscript:

ADAs	Anti-drug antibodies
ADHD	Attention-deficit/hyperactivity disorder
AE	Adverse effect
AOMs	Anti-obesity medications
BED	Binge eating disorder
BMI	Body Mass Index
CBT	Cognitive–behavioral therapy
CI	Confidence interval
COVID-19	Human coronavirus disease 2019
EE	Emotional eating
GIP	Glucose-dependent insulinotropic peptide
GLP-1	Glucagon-like peptide-1
GLP-1R	Glucagon-like peptide-1 receptor
*GLP1R*	Gene encoding the glucagon-like peptide-1 receptor
GLP-1RA	Glucagon-like peptide-1 receptor agonist
GoF	Gain-of-function
HbA1c	Glycated hemoglobin
HPA	Hypothalamic–pituitary–adrenal
HPO	Hypothalamic–pituitary–ovarian
IBT	Intensive behavioral therapy
LCD	Low-calorie diet
LEPR	Leptin receptor
LH	Luteinizing hormone
LoF	Loss-of-function
MAO	Metabolically unhealthy obese
MASLD	Metabolic dysfunction-associated steatotic liver disease
MC4R	Melanocortin 4 receptor
*MC4R*	Gene encoding the melanocortin 4 receptor
MD	Mean difference
MeSH	Medical Subject Headings
MHO	Metabolically healthy obese
MNT	Medical nutrition therapy
MONW	Metabolically obese with normal weight
MRI	Magnetic resonance imaging
MUO	Metabolically unhealthy obese/obesity
Nabs	Neutralizing antibodies
NES	Night eating syndrome
NHS	The National Healthcare Service (England)
NMRI	Nuclear magnetic resonance imaging
PCOS	polycystic ovary syndrome
PCSK1	Proprotein convertase subtilisin-kexin type 1
POMC	Proopiomelanocortin
PSQI	Pittsburgh Sleep Quality Index
RCT	Randomized controlled trial
RYGB	Roux-en-Y gastric bypass
SAT	Subcutaneous adipose tissue
SCFAs	Short-chain fatty acids
SG	Sleeve gastrectomy
SNAC	Salcaprozate sodium
SO	Sarcopenic obesity
SR	Sustained-release
SSRIs	Selective serotonin reuptake inhibitors
VLCD	Very low-calorie diet
